# Assessing Kurt Goldstein’s lasting influence in the neuropsychology of language versus his use of aphasic symptoms as diagnostic insights into brain injuries

**DOI:** 10.3389/fpsyg.2024.1356824

**Published:** 2024-08-22

**Authors:** Frank W. Stahnisch

**Affiliations:** ^1^Department of Community Health Sciences, University of Calgary, Calgary, AB, Canada; ^2^Department of History, University of Calgary, Calgary, AB, Canada; ^3^Hotchkiss Brain Institute, Cumming School of Medicine, University of Calgary, Calgary, AB, Canada; ^4^O’Brien Institute for Public Health, Cumming School of Medicine, University of Calgary, Calgary, AB, Canada

**Keywords:** aphasiology, Norman Geschwind, Kurt Goldstein, history of neurology, holism and localizationism, Carl Wernicke

## Abstract

In the history of the neurological relationship between human behavior and brain function in Europe and North America, various perspectives on brain localization and holistic functioning have been addressed. One of the founding figures of modern neuropsychology, Professor Hans-Lukas Teuber (1916–1977) of the Massachusetts Institute of Technology, reminded the scholarly community of its negligence of preceding traditions in day-to-day research endeavors. Teuber particularly emphasized that during the development of the aphasiology field (1950s–1960s) even major figures, such as the German-American neurologist Kurt Goldstein (1878–1965), had been neglected in the scientific community’s collective memory. This happened despite Goldstein’s contributions to cortical blindness, vicarious brain functioning, and neurorehabilitation. The outcome of the debates regarding the neurology of language had to be incompletely relearned in later decades. Neuropsychological concerns regarding the relationship between cortical localizationism and functional holism have made recourse to Goldstein’s work necessary for reviving historical answers for current conundrums. It is therefore opportune to review Goldstein’s work in the light of the history of aphasiology. Contemporary scholarship has once more drawn research attention to the works of Goldstein along with Norman Geschwind (1926–1984) and his pupils. It has also resurrected the underlying research of Carl Wernicke (1848–1905). This review article explores deep and lasting questions regarding the positioning of Goldstein’s holism among the contemporary holistic perspectives. It does so by firstly discussing Wernicke’s traditional model of distributed localizationism. Secondly, it describes Goldstein’s previous work in the German brain sciences. Thirdly, it examines his aphasiological contributions on both sides of the Atlantic. Fourthly, it addresses the advancement of a dynamic localizational perspective by Geschwind and his pupils. This article intends to render a historical analysis fruitful for those exploring modern-day problems in the neurology of aphasia and clinical speech neuropsychology.

## 1 Introduction

There is a widespread view in the scholarly community that the development of scientific ideas, research procedures, and therapeutic approaches in neurology and psychology follows a steady process of knowledge accumulation in the aphasiological field (Marx, 1966; Kushner, 2015). It could thus appear trivial to search exclusively for a continuous stream of ideas that advanced with continual adjustments toward scientific betterment. In 1964, the Boston-based founding figure of the neuropsychology of language and aphasiology, Norman Geschwind, reviewed the history of aphasiology in a widely received article entitled “The Paradoxical Position of Kurt Goldstein in the History of Aphasia.” He presented the German-American neurologist as a mediating figure between the faction of cerebral localizationists and the opposing faction of holists in regard to higher neurophysiological functioning (Geschwind, 1964, pp. 214–218). This perspective can be seen as stemming from his work as a neurologist with an “incisiveness of his clinical perception and the infectiousness of his wit,”—one who observed patients with acute focal brain injuries and conceived the view that the localization of language functions was, indeed, a robust clinical phenomenon. He thereby resurrected the concept of functional-anatomical localization that had been fading in the early 1920s due to the rise of anti-localizationism in neurology. This tension between localization versus holism, however, was only resolved in the decades following Geschwind’s death (Waggoner, 1984, p. 27).

Particularly Geschwind’s work on classification and categorization of aphasic symptoms is of importance here since he characterized Goldstein as clinging to the Wernickian localization approach to aphasia while distancing himself in other areas of neuropsychology and behavioral neuroscience (De Bleser, 1994, pp. 319–347). Dr. Goldstein, an academic refugee to North America, had himself been ousted from his position as a neurology professor at the Charité Medical School in Berlin in 1933, needing to flee Germany the same year to save his life. Passing through stages of his exile in Zurich, Switzerland, and Amsterdam, The Netherlands, he arrived in the US in 1935. Taking his protracted path of emigration into account, Goldstein’s pioneering role in clinical cerebral localization, and as a founding figure of aphasiology and neurorehabilitation, appears to have been fully dependent on the side factors of his ergobiography. For this remarkable physician and scientist, those factors included his flight, persecution, and jarring re-adjustment to the medical world of North America (Bach, 1958, p. 93).

In Geschwind’s article, he expressed that Goldstein’s influence on the neuropsychology of language and of aphasiology would be for decades to come. He had altered the received academic viewpoints and added innovative holistic insights that allowed for an analysis of the more basic substrates of higher cognitive functions in the United States (US), but also in the United Kingdom (UK), after WWII:

“The work of Kurt Goldstein is particularly discussed, and it is pointed out that it is readily apparent from Goldstein’s own works that he accepted a majority of the classical teachings, indeed even in details. It is also pointed out that many of Goldstein’s holistic theoretical views were in fact so extensively qualified as to make them compatible with almost any approach. There is some discussion as to the reasons for the widespread rejection of the classical views when even its apparently severest critics accepted so much of the classical teachings” (Geschwind, 1964, p. 224).

With this observation about the state of neuropsychology, Geschwind circumvented the existing tensions between traditional localizationist perspectives on aphasia since the 19th century and the modern approaches which emerged at the beginning of the 20th century (Aminoff et al., 2008, p. vii).

In light of Geschwind’s nuanced comment, this article assesses Goldstein’s lasting influence in the neuropsychology of language versus his use of aphasic symptoms as diagnostic insights into brain injuries. Moreover, it applies a specific view to Geschwind’s assumption that Goldstein’s holistic theoretical views had been compatible with most approaches in clinical neurology and language neuropsychology. After establishing that Goldstein was indeed a critical figure in the history of aphasiology, his experimental and important diagnostic work with German Gestalt psychologist Adhémar Gelb (1887–1936) on classification-categorization and neuropsychology will be examined. After clarifying this early background on the pathologies of speech production and language comprehension, Geschwind’s ambivalent conceptual take on Goldstein’s aphasiology will be investigated in order to amalgamate more recent holistic interpretations of aphasia with Wernicke’s approach. This helps to distance Goldstein and his coworkers’ research stances from other scholars (LeBlanc, 2021, p. 277). Especially, his collaborative work on brain plasticity with Frankfurt-based physiologist Albrecht Bethe (1872–1954) can shed some additional light on Goldstein—his views representing the diversity of aphasia (s)—as a mediating figure between the holists and localizationists (Stahnisch, 2016, p. 2).

**Figure 1:**
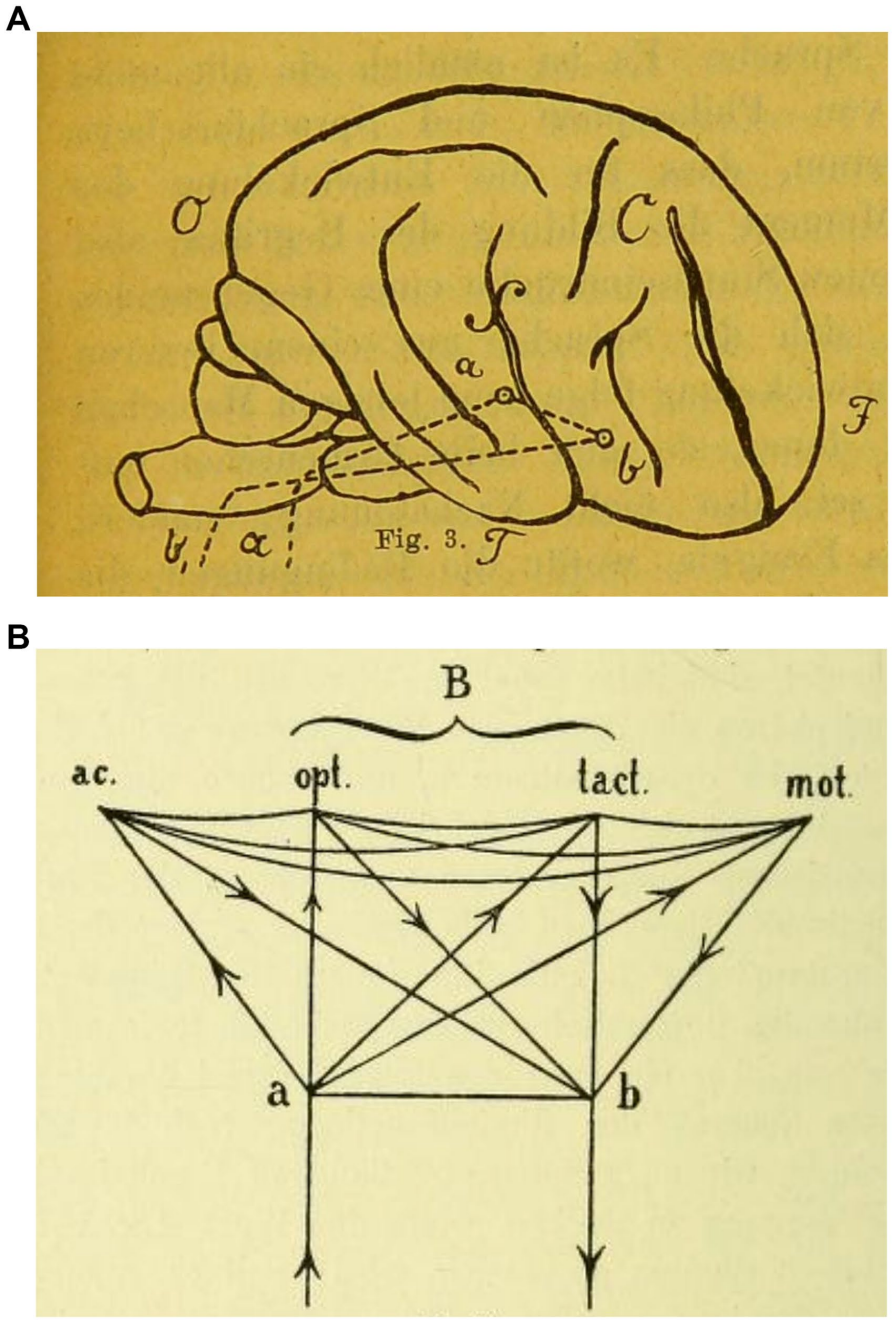
**(A)** Wernicke (1874), *Der Aphasische Symptomenkomplex. Eine psychologische Studie auf anatomischer Basis*, 19. Sketch © Public Domain. **(B)** Wernicke (1893), *Gesammelte Aufsaetze und kritische Referate zur Pathologie des Nervensystems*. Berlin, Germany: Fischer, 100. Sketch © Public Domain.

The positions of the early Frankfurt-and Berlin-based brain scientists that they had developed based on clinical analyses of subcortical aphasias in brain-injured soldiers from WWI represented a further departure from Wernicke’s traditional localizational view (Figure 1). It needs to be pointed out that the above figure, which Wernicke had primarily designed to describe the main physiological process of speech production (“*Entwicklung des Sprachvorgangs*”), has been simultaneously used to depict localizational concepts in aphasia. Although it certainly formed the basis of Wernicke’s general functional views towards the production of language and speech, it was also employed by holists when they deplored the views of the “diagram makers” (Eggert, 1977, p. 180). Nonetheless, he intended this early diagram primarily to illustrate his concept that the very basic aspects of language and other higher brain functions could be localized in a gross anatomical form (Wernicke, 1874, p. 18). Wernicke’s fuller—and specifically pathophysiological—views on the neurology of language were laid out in his 1874 monograph (Figure 1) and elaborated in follow-up publications (Wernicke, 1893). This notion was ultimately blueprinted in a later article on “Some Recent Works on Aphasia” (Wernicke, 1885; Wernicke, 1886) as a complex assortment of nerve centers—something which he had first conceived from the ideas of Ludwig Lichtheim (1845–1928) at the University of Koenigsberg in East Prussia and further elaborated upon (Eling, 2011, pp. 501–508). Wernicke wittily referred to this diagram as a central telegraphy corporation (a “*Centraltelegraphenanstalt*”)—following an earlier concept by Vienna neurophysiologist Sigmund Exner (1846–1926) (Wernicke, 1874, p. 220). Elliott D. Ross has interpreted Wernicke’s concept as something akin to a complex ‘neural network’ that transcended the foregoing reflexive flowchart of the basic functional language processes (Ross, 2010, p. 224). Thus, Wernicke’s Figures 1A,B can be seen as complementary to one another, the one on top showing the gross anatomical substrates and the one at the bottom representing the complicated and, as he claimed, hidden processes (“*complicirte Vorgaenge*”) in the neurology of language.

As Geschwind claimed, this mediating stance was likely not very elaborate, but it certainly provided a very useful step towards establishing the field of the neurorehabilitation of aphasic disorders already in the 1910s and 1920s (Frommelt, 2015, p. 353).

## 2 The prehistory: Kurt Goldstein in Frankfurt and Berlin

Born into a Jewish family in the German province of Silesia, Goldstein first studied philosophy in Heidelberg and later shifted to the study of medicine at the University of Breslau where he graduated M.D. in 1903 (Goldstein, 1903). His morphological thesis, conducted in the Psychiatric Clinic of Wernicke, examined the structure of the posterior columns of the spinal cord (Eggert, 1977, pp. 7–8, 50). Goldstein’s philosophical outlooks were influenced by his brother-in-law, the Hamburg cultural philosopher Ernst Cassirer (1874–1945)—something which fed into his detailed structural analysis of the interplay of form and function in physics, biology, and clinical anatomy (Pow and Stahnisch, 2014, p. 1049). Between 1906 and the outbreak of WWI, Goldstein completed his residency in neurology and psychiatry at the University of Koenigsberg in East Prussia where he graduated for a second time, his *Habilitation* thesis having been carried out in Ernst Meyer’s (1871–1931) neuropsychiatric clinic.

Over a period of years, the eminent Frankfurt neuroanatomist Ludwig Edinger (1855–1918) became aware of him and his particular work on language disorders that followed brain injuries in the human cortex (Stahnisch, 2008, p. 148). Edinger offered Goldstein the directorship of the Institute for Research into the Effects of Brain Lesions (“*Institut fuer die Erforschung der Folgeerscheinungen von Hirnverletzungen*”) during WWI so that he could join the experimental psychologist Gelb who, indeed, went on to direct the neuropsychological service there until 1930 (Stahnisch, 2024, pp. 113-117).

**Figure 2:**
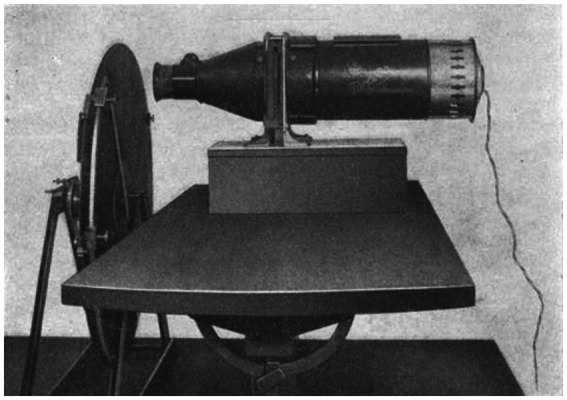
Tachistoscopic vision defect experiment at Goldstein’s and Gelb’s Institute for Research into the Effects of Brain Lesions: Goldstein and Gelb (1918), Psychologische Analysen hirnpathologischer Faelle auf Grund von Untersuchungen Hirnverletzter, fig. 48, p. 141. Photograph © Public Domain.

It was in the clinical subdivision of Edinger’s Neurological Institute (integrated into the new and privately endowed University of Frankfurt am Main in 1914) that their collaborative work ensued. This “Institute for Research into the Effects of Brain Lesions” had been established as part of the medical preparations—in 1916—for the expectedly fierce Battle of Verdun on the Western Front. It remained in operation throughout the Weimar Republic until the Nazis seized power in 1933. There, Goldstein’s and Gelb’s working relationship and mutual exchanges revealed the crystallization process of fruitful interdisciplinary and methodical work in experimental neuropsychology (Figure 2). While Goldstein had a much more philosophical attitude and broader neurological interest, it was Gelb who contributed refined technical approaches for succinct and analytic laboratory experimentation on human subjects that led to a prolific scientific output (Teuber, 1966, pp. 301–302). Goldstein and his coworkers, among them the experimental physiologist Albrecht Bethe, closely fostered research ties with many of the affiliated units and institutions in the city of Frankfurt between 1916 and 1930 (Stahnisch, 2016, pp. 2–5). In 1929, Goldstein was even designated as Edinger’s successor to direct the multidisciplinary Frankfurt Neurological Institute:

“My idea was to build an institution which offered the opportunity to observe the patients’ everyday behavior and to study them in all respects. Accordingly I organized in Frankfurt am Main, under the administration of the government, a hospital which consisted of a ward for medical and orthopedic treatment, a physiological and psychological laboratory for special examination of the patients and theoretical interpretation of the observed phenomena, a school for retraining on the basis of the results of this research, and finally workshops in which the patient’s aptitude for special occupations was tested and [the patient] was taught an occupation suited to his ability. I was assisted in this work by younger neurologists, teachers, and psychologists. […]” (Goldstein, 1971, p. 3).

Unfortunately, Goldstein was not granted the funding for a proper psychiatric ward at the Institute because the service had been divided up by the university administration. The directorship for the psychiatric clinic was instead given to Karl Kleist (1879–1960), a development which stirred up contention and great disappointment for Goldstein. As a result, he left Frankfurt in 1930 for Berlin where he obtained the directorship of the Clinic for Neurology at the Charité teaching hospital in Moabit. There, Goldstein was very strategic in choosing a group of accomplished, young, and innovative experts for his service (Harrington, 1996, pp. 103–139). Between 1931 and 1932, the Jewish neurohistologist Karl Stern (1906–1975), formerly employed in Goldstein’s service in Frankfurt, was running a high level of neurohistological practice in Moabit. Its basic and clinical research facilities rendered the institution soon to be one of the renowned city hospitals in Germany. It hosted Ernst von Bergmann’s (1836–1907) pupil, Moritz Borchardt (1868–1948), as a versatile neurosurgeon. Adhémar Gelb was the long-term experimental psychologist and, for a short period, Ludwig Pick (1868–1944) joined on as the staff neuropathologist. At the time, Moabit was the only academic hospital, out of the few that existed in Berlin, with distinct services in neurology, psychiatry, and pathology (Stahnisch, 2020, pp. 124–125). It was there that Goldstein made numerous and progressive contributions to the fields of brain psychiatry, clinical neurology, experimental psychology, medical rehabilitation, and philosophical anthropology. He and his group tried to combine the analytical approach of classical neurology with a holistic theory of brain functioning that integrated the insights of contemporary Gestalt psychology. In his clinical departments in Frankfurt and Berlin, Goldstein educated many cohorts of medical students not only in basic brain research and neuropathology but broader psychoanalytic and clinicopathological approaches (Goldstein, 1934, p. 131).

He undoubtedly had plans to develop the facilities at Moabit hospital to reflect his closely held ideas of a holistic model of the brain sciences in an organic form. Yet, this did not come to complete fruition since, just when everything was institutionally set for Goldstein’s clinic to develop into one of the major centers of German neurology, a catastrophe ensued (Geroulanos and Meyers, 2018, p. 106). Nazi leaders saw in Moabit’s hospital a “reddish” and “Jewish” institution. It soon became the target of brown mobs after Nazi writers attacked Goldstein in an article, published on the 21st of March 1933 in the newspaper “*Der Stuermer,*” for being a Jewish physician in a high medical position—a neurologist primarily concerned with therapy rather than the societal exclusion of the mentally and neurological ill (Pross and Winau, 1984, pp. 184–186). The situation deteriorated and waves of expulsions ran through the country, driving socialists, communists, democrats, and Jews out of their positions and into internment camps. The attacks and criticisms on Goldstein were unleashed even before the infamous “Law on the Reestablishment of a Professional Civil Service” was passed on April 7th, 1933. This law determined that state officials—understood as being individuals “of non-Arian descent”—were to be dismissed from office. This development meant a deep severing within the established culture of science and medicine in Weimar Germany (Kater, 1989, pp. 111–126).

Stormtroopers arrested Goldstein while he was attending patients in his clinical service, and he was already incarcerated on April 1st, 1933. Being a prominent member of the “Union of Socialist Physicians,” Goldstein was tortured in the Berlin prison in *General-Pape Street*. Only owing to the pleas of his former student and future wife, Dr. Eva Rothmann (1878–1960), the prominent Nazi psychoanalyst Matthias Heinrich Goering (1879–1945) intervened on Goldstein’s behalf. He was released from prison but had to sign a pledge that he would leave Germany forever. He fled to Zurich, Switzerland where he co-founded the “Union of German Scientists in Despair” (“*Notgemeinschaft der Deutschen Wissenschaft*”) with the Catholic novelist Carl Zuckmeyer (1896–1977) and the Turkish émigré pathologist Philipp Schwartz (1894–1977) (Schwartz, 1995, pp. 42–45).

## 3 Goldstein’s work on categorization and neuropsychology on both sides of the Atlantic

Eventually Goldstein found refuge in the Pharmacological Institute of Amsterdam, mediated by the Dutch neurologist Bernard Brouwer (1881–1949). There he could finish his seminal publication “The Architecture of the Organism” (*Der Aufbau des Organismus*) with the help of the Rockefeller Foundation which had initiated an aid program of $60,000 to support refugee-doctors and scientists in leaving Germany (Schneider, 2002, pp. 208–222). In 1935, Goldstein emigrated to New York City, where he continued clinical work as a neurologist in his private practice while lecturing between 1937 and 1938 at Harvard University and teaching at Columbia University until the end of WWII (Goldstein, 1940). Yet, having lost his organizational basis and the group of highly skilled co-workers through his forced migration from Nazi Germany, Goldstein, who was already 57 years old, did not succeed in re-establishing an interdisciplinary department or even a diversified working group in the US. Being forced to practice for a living, while lecturing at diverse institutions, was demanding on him. He was obliged to stretch his research interests into psychology and sociology, diverting attention away from clinical neurological research (Geroulanos and Meyers, 2018, p. 106). This is reflected in a description provided by Harvard psychologist Marianne L. Simmel (1923–2010) who met Goldstein in 1942 in Boston and judged his scientific career as a clinical neurologist to have been ruined by forced migration. Americans simply could not understand what he was intending to be: “they asked: a physician, a psychologist or a philosopher?” (Simmel, 1968, p. 11). As she pointed out, the socialist Goldstein did not request sufficient money from many of his poor patients during an economic depression and plunged himself into financial hardship too. He did not find the adequate context and suitable scientific culture that he was seeking nor did not feel right at home, mainly because the close relationship to his own culture had been disrupted. This was reflected in Goldstein’s Harvard lectures:

“Ultimately all failures [including scientific failure] in social organization are caused by an underestimation of the significance of the abstract attitude and by the misjudgment of the detrimental influence which can emanate from human traits if one changes them through artificial isolation. With the help of the abstract attitude the fallacy which is basic to all false social organization can be disclosed” (Goldstein, Lect. 9, 1940, pp. 223–224).

Although fervently searching for the cognitive abstract and categorical attitude—here used in the context of social adaptation to new environments—Goldstein was quite aware of the difficulties this entailed. His group of émigré pupils and collaborators—including the Montreal neuropsychiatrist Karl Stern and the Richmond, Virginia-based neurologist Walther Riese (1890–1976)—never fully realigned during their forced migration to North America. Not even in Wilder Penfield’s (1891–1976) Montreal Neurological Institute or the large research centers at the University of Virginia was such a development possible. Stern had been obligated to emigrate to London in 1939 before finding exile in Canada (Stern, 1951, pp. 97–80). These clinical researchers’ interests in holistic neurology and the psychological cognitive defects went much further than was permitted in the narrow programs and routine cultures of the specialized centers they encountered in the US or Canada. Like Goldstein, who had shifted from holistic neurology to applied experimental psychology, Riese was no longer working as neuropathologist, venturing instead into theoretical neuropsychology and later medical history (Riese, 1953). His book, *The Conception of Disease*, consists of a scholarly account of the conceptions of disease throughout the ages with particular reference to the different concepts in diverse historical cultures. The extent of their problem was perhaps best highlighted in Riese’s discussion of disease as an essential concomitant of human development. It is only when the existence of disease is accepted that further neurological and cognitive development becomes possible, a position which his mentor Goldstein had also endorsed when alluding to the “vicarious functioning of the human nervous system” as a realization in a brain disease context (Riese, 1968, pp. 25–27).

Among Goldstein’s great losses was the death of his former psychologist Gelb after being expelled from his chair at the University of Halle and not surviving long enough to take the professorship in experimental psychology which Kansas State University had offered to him:

“It was primarily through the close conjunction of Goldstein the neurologist with Gelb the psychologist that neuropsychology flourished in the Frankfurt Institute. The deep friendship between the two men is a testimony to their character. They were magnificently complementary in training and temperament, each capable of transmitting to the other much of his special skill. Their collaboration exemplifies the division of labor which occurs even in the smallest social system […]: Goldstein had much the firmer grasp of general neurology together with clinical intuition and a sense for broad questions; he found it easy to write, while Gelb was more of the experimenter […]. It is remarkable how the relationship between these two men remained free from the strains of diverging orientations or personal ambition; their partnership ended only with Gelb’s premature death in 1935 when he and Goldstein had just left Germany for Holland where they were jointly awaiting their U.S. visas which were to bring them to America on Rockefeller fellowships” (Teuber, 1966, pp. 301–302).

As becomes clear with a view to the medical situation in the aftermath of WWI and the socio-political dimensions of the Weimar Republic, the once-glowing aura of holistic concepts in medicine and psychiatry had dimmed. Discourse on neurodegeneration had shifted from traditional perceptions of clinical problems regarding the rehabilitation of the war wounded and investigations of underlying neuropsychological processes. After he arrived in North America, Goldstein described the categories of his test approaches in the preface to *After Effects of Brain Injuries in War* (Goldstein, 1942), deciding to share his earlier work treating war veterans as a clinical neurologist with his new neuropsychology colleagues in his host country. He thereby emphasized that work with acute clinical and exclusively somatic patients was very different from the work setting as a neurologist who often looked at the longer-term effects of war injuries. He stated that he had experienced his former approaches to patient and symptom categorization and neuropsychological research as being much more centered on rehabilitation demands than immediate cure and recovery. This perspective also had implications for Goldstein’s diagnostic practices and application of neuropsychological tests (Eling, 2017). For example, he continued to develop behavioral tests that looked at concept formation (stemming from earlier experiences with aphasic and mute brain-injured veterans) as non-verbal quantified types that merged into the Wisconsin Card Sorting Test and similar test approaches used in the neuropsychological laboratory. Furthermore, Goldstein added to clinical assessment strategies by insisting on analyses of task and work performance as well as explorations of cognitive dysfunctions through situational variations of experimental and clinical tasks (as he and his group had done earlier in the hospital wards in Frankfurt and Berlin) (Teuber, 1966, p. 306).

## 4 Goldstein’s aphasiology and rehabilitation work

Interdisciplinary exchanges in brain science had been emphasized in the Goldstein group since its early days in the Frankfurt Institute for Research into the Effects of Brain Lesions. There, they had studied the psychological, philosophical, and neurological interchanges of brain functioning in a broad clinical and rehabilitation-oriented manner. Clearly, WWI marked a watershed moment for the practice of neurology in the 20th century (Linden and Jones, 2023, p. 628). For a long time, Berlin neurologist Hermann Oppenheim (1958–1919) had been the only prominent advocate of the view that structural neural changes underlay war-related traumata. However, by the end of the war, up and coming neurologists and physiologists like Goldstein, Bethe, and Riese had jumped on the same bandwagon and helped creating the new field of neuro-traumatology. Moreover, the search for a “psychopathic constitution” or a “degenerative disposition” of the brain noticeably accelerated after 1919 when it became clear that neuropsychiatric conditions among war-injured veterans were often of a subjective and fluid nature (Schmiedebach, 1999, pp. 27–30).

It was not merely a yearning for fair health insurance and compensation that was at stake during this time; the German State invigorated a postwar occupational sense of therapy and social recovery that equated the health of the body with the ability to work. Health care administrator Hermann Hartmann (1863–1923), who inaugurated the first meeting of the German Medical Association, wrote a seminal manual for welfare departments and their evaluating physicians that provided detailed advice about health care. It asserted that all disabled veterans and their families should embrace the idea of national healing through mutual work efforts:

“The war wounded, and their dependents have suffered exceptionally under the nerve-shattering effects of the world war: their speech, their movements, their ability to feel, their inner being has been fundamentally changed by today’s murderous torments to the body and spirit.” (Hartmann, 1919, pp. 32–33).

War victims were depicted as alienated from their earlier work contexts; they had to be fully reconnected with the new German society after the war. Yet, such a social democratic view of labor relations was in many ways romantic, and it became soon undercut by the economic turmoil which sheared the liberal roots of the Weimar Republic. The contemporary images of men physically tied to their workbenches and machines resonated strongly with the communist philosopher Karl Marx’s (1818–1883) prediction that humans of the future would become nothing more than appendices to machines. Such images also offered hope that neurorehabilitation would provide the war-injured with the tools and practices for their renewed social participation:

“In the brain-injured patient, we encounter changes of structure. Resulting from this, a whole series of earlier and normal action-realizations [*Reizverwertungen*] can no longer be sustained, and previous viable tasks no longer resolved. When the patient is confronted with them, however, various abnormal action-realizations occur, which I call ‘catastrophic reactions’ [*Katastrophenreaktionen*]” (Goldstein, 1927a, p. 238).

Soviet neuropsychologist Alexander R. Luria (1902–1977) was invited by French neuropsychologist and editor-in-chief Henry Hécaen (1912–1983)—who had collaborated with Geschwind since a sabbatical period in Boston—to contribute to a special issue of the journal *Neuropsychologia* (Whitaker, 1985, p. 138). In it, Hécaen had gathered the contributions of leading authorities to write about their experiences with, and assessment of, Goldstein’s work and academic role in the US. As Luria has pointed out, Goldstein—with his concept of the “catastrophic reactions”—tried to combine the analytical approach of classical brain science with the holistic theory of cortex functioning and the higher psychological processes, all within the framework of contemporary Gestalt theory. Goldstein’s earlier views on the neurology of aphasia were based on the observation that in aphasic states, there could be partial lesions of, for instance, both the anterior and posterior speech areas, and thus not singular destructions, which led to “mixed-transcortical” or “isolational” forms of aphasia (Goldstein, 1915, pp. 45–47). Clinically, these led to behavioral phenomena such as echolalic repetition and a reduction of speech output. Such concepts differed notably from his later views in which Goldstein resorted to the concept of “trans-cortical sensory aphasia.” This would help in addressing clinical concerns with the expressive side as separate from concerns with the perceptive side of language production (Goldstein, 1948, p. 151). Viewing them as a “motor symptom complex” (*ibid.*, 293) allowed for a more distributed and multidimensional understanding of aphasias when, for example, observing patients with fluent speech and a preserved faculty to repeat, who nonetheless possessed poor comprehension of what was spoken to them. In contrast with the foregoing unidimensional view of brain functioning, Goldstein (1925a) stated “that the symptom cannot be regarded as an immediate expression of the damaged function: it has to be analyzed, and only an analysis of the basic disturbance which has to be singled out can show its real essence; this basic disturbance can solve the riddle of the whole syndrome—and only when it becomes clear is the clinical analysis of the patient over” (Luria, 1966, p. 312). Goldstein’s refined theories and methods assumed that the nervous system acted as a network, mediated by ganglia (“nerve knots”), and reacted in its totality to the outside world through the sensorium and motor action:

“Every stimulus which affects this consistent apparatus – this ‘system’ – generates a modification of the whole apparatus. This modification finds it external expression in movements of the target organs. […] The organism exists only in its own milieu; this means only those things in the outside world which are capable to merge with the system of the organism into a more extensive system get ‘captured’ by the organism [and] constitute its milieu. All others actually don’t exist. If they enforce access, they affect it as disturbances, the impact of which either has to be eliminated or leads to severe dysfunctions in the whole system of the organism […]” (Goldstein, 1925b, p. 376; author’s transl.).

Luria noted that the method of psychologically qualifying the neurological phenomena which Goldstein introduced in a short article from 1925 (“The symptom, its emergence and meaning for our view of the building and of the function of the nervous system”) marked the beginning of modern neuropsychology. Goldstein himself called his approach retrospectively “a kind of philosophical anthropology” (Goldstein, 1971, p. 12).

**Figure 3:**
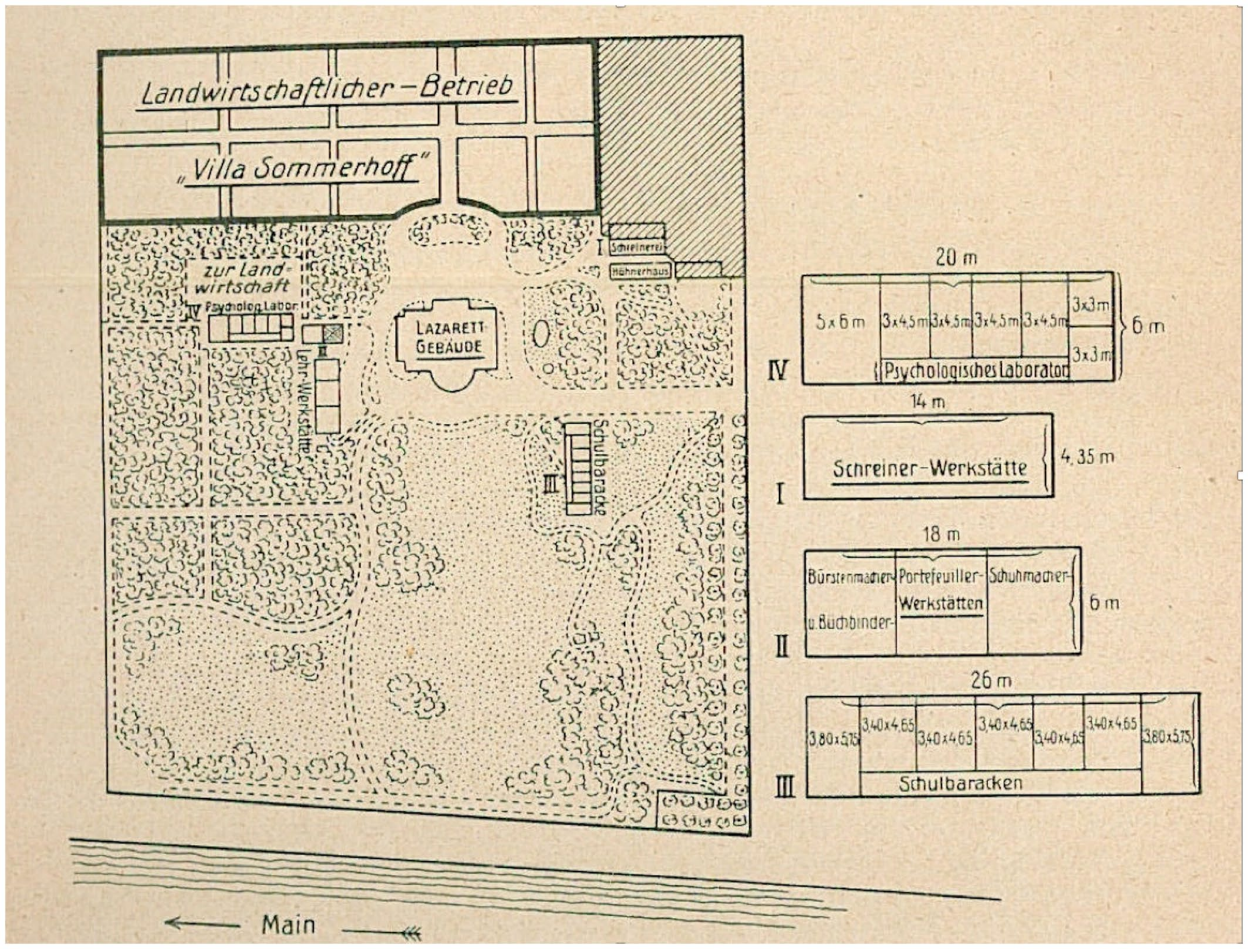
Map drawing of the Villa Sommerhoff with adjacent functional buildings of the rehabilitation unit: Goldstein (1919), *Die Behandlung, Fuersorge und Begutachtung der Hirnverletzten. Zugleich ein Beitrag zur Verwendung psychologischer Methoden in der Klinik*, 3. Map © Public Domain.

The work performed between 1916 and 1930 by Goldstein and his interdisciplinary group at the Institute for Research into the Effects of Brain Lesions (Figure 3) can be seen as a particularly good example for researchers seeking to closely study the cultural interrelations between neurology through the integration of philosophy of mind, social psychiatry, and scientific innovations that emerged through “holistic neurology.” It would, however, be erroneous to regard their program as a monolithic research tradition since it displayed ambiguities even within contemporary neuropathological views. Prominent examples were Goldstein’s experiments on “The physical constitution of the brain” in his collaborations aimed at speech recovery, treatment of apraxia, and management of visual impairments. In fact, the social and cultural impact of WWI gave a sense of urgency to the research and efforts of many holistic physicians and psychologists. Goldstein later affectionately recalled this to Edinger, stating: “Your work with human beings is of much greater importance than my theoretical work in the laboratory” (Goldstein, 1971, p. 5).

With regard to clinical and neuropsychological research within its walls, the so-called Villa Sommerfeld (which housed the Institute for Research into the Effects of Brain Lesions) was acclaimed for the vigorous rehabilitation efforts taking place there. They were designed after the latest principles of work science and through close interchanges with the Frankfurt physiologist Bethe and the members of his institute of physiology. The neurorehabilitation approaches used there stood in stark contrast to the woefully inadequate therapeutic measures and research approaches in mental asylums (*Nervenkliniken*) and penitentiaries (*Arbeitshaeuser*) in Central Europe (Hoffmann, 2021, pp. 191–208). According to Goldstein’s own description of Villa Sommerfeld:

“It consisted of a ward for medical and orthopedic treatment, a physiological and psychological laboratory for special examination of the patients, and theoretical interpretation of the observed phenomena, a school of retraining on the basis of the results of this research, and finally workshops in which the patient’s aptitude for special occupations was tested and he was taught an occupation suited to his ability” (Goldstein, 1971, p. 3).

Most promising was their program for the rehabilitation of brain-injured patients, aphasics, and the neurologically paralyzed. It was initiated immediately after brain surgery and wound closure as described in the important 1919 report on *The Treatment, Care, and Evaluation of the Brain-Injured*. There, Goldstein stated that 63% of the patients had been able to return to their prewar positions, 17% could commence albeit in a new work assignment, only 10% percent remained unemployed, and another 10% had to remain hospitalized (Goldstein, 1919, p. 3).

His neurorehabilitative work, as mentioned previously, would have been unfeasible without the help of Gelb who designed the new psychological tests for the returning war-injured and later helped develop the research program on language disorders, motor deficits, and visual impairments (Ackermann, 2023, pp. 142–168). With regard to contemporary debates around the physical constitution of the brain, Goldstein’s experiments with Gelb and Riese on behavior improvements in young soldiers began to raise doubts about the process of functional integration and regeneration in the human nervous system. Most notable was the case of the 24-year-old patient, Schneider, who had two lesions in the posterior portion of his brain (i.e., the visual cortex). Goldstein and Gelb realized that uninjured parts of Schneider’s brain must have taken over functions from destroyed parts of his brain (Eling, 2015, p. 328). To the researchers, this meant that the brain appeared to have adaptive capacities even in adult patients. They noted that Schneider was still able to read any text through “a series of minute head and hand movements; he ‘wrote’ with his hands what his eyes saw […]. If prevented from moving his head or body, the patient could read nothing whatsoever” (Goldstein and Gelb, 1918, p. 124; author’s transl.).

**Figure 4:**
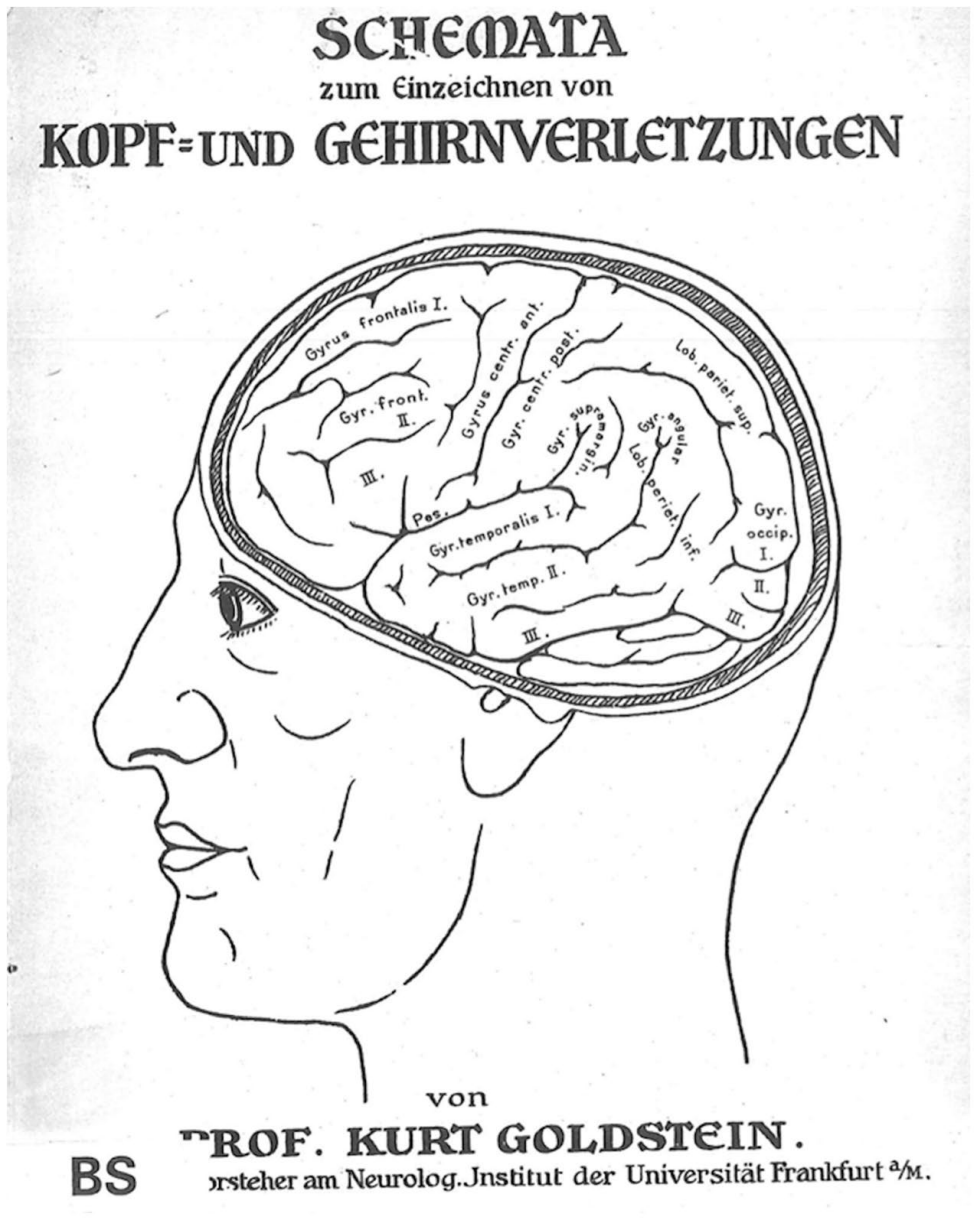
Schemata of head and brain injuries for clinical-diagnostic purposes: Goldstein (1916), *Schemata zum Einzeichnen von Kopf-und Gehirnverletzungen*, Cover. Sketch © Public Domain.

This finding meant that body movements partly compensated for cognitive functional loss, and the physical constitution of the brain played an important part in the recovery of functional impairment. In light of their clinical neurophysiological and linguistic adaptational work, this could not have been the full story: When investigating language recovery, cognitive disorders, and their interrelation with practical skills, Goldstein and his collaborators began to realize that the physiological and psychological effects of the “catastrophic reaction” in the brain could rule out anatomical destruction phenomena. He and his coworkers nevertheless held the conviction that more needed to be learned about the brain’s regeneration, adaptability, and the structure–function relationship than had so far been gathered in the clinical neurology of his time (Figure 4).

Goldstein and his colleagues found that similar physiological and behavioral symptoms could be associated with fairly different structural origins, and inadequate treatment was often applied. The same patient, unable to achieve the task when commanded, “Close your eyes!” could nevertheless be able to execute it upon being asked to act out falling asleep. A patient with a lesion of the cerebellum who was unable to point with his finger to the tip of his nose was still capable of grasping his nose (Goldstein, 1931, p. 454). The experimenters discovered that there were at least two different groups of symptoms. The first group included symptoms that related to concrete behaviors in everyday situations such as turning on a light switch or saying “Hello” at a reception. These behavioral traits were more or less unconscious, unreflective, and lacking sense of situational belonging. The second group included symptoms which pertained to abstract behaviors such as awareness, reasoning, and reflecting on one’s own actions (Goldstein, 1927b, pp. 600–602).

With a specific view to the socio-political dimensions of the Weimar Republic, the primary focus on holistic concepts in medicine and psychiatry waned as a result of having to provide room for contemporary discourses about neurodegeneration and reductive anatomical lesion loads. These discourses shifted away from traditional perceptions of clinical problems of casualty rehabilitation and investigations into the underlying structural and causal processes. Yet with their holistic emphasis on rehabilitation, Goldstein’s collaborators pursued a line of medical research that, according to historian of science John Cornwell,

“was generally criticized by Nazi doctors for its ‘negative features’, which were described as ‘liberalism, individualism, mechanistic-materialist thinking, Jewish-communist human ideology, lack of respect for the blood and soil, neglect of race and heredity, emphasis on individual organs and the undervaluing of soul and constitution’” (Cornwell, 2003, p. 154).

Persecution and flight to other countries, such as the Netherlands, France, England, Canada, and the USA were solid reasons for the Goldstein group’s later development into an excessively loose network. It not only lost its strong interdisciplinary ties which it once had in Frankfurt and Berlin, but with its dissemination to New York, Montreal, Maryland, Kansas, and Richmond, it could no longer uphold its previous momentum. The members of the group encountered a different research landscape in Canada and the US which was moved by incongruous scientific goals that showed a lack of appreciation for the integration of neurology with philosophy, psychology, and rehabilitation. That very integration was the program that the holistic neurologists embraced in the past (Ludwig, 2012, p. 52). As Goldstein’s biographer Simmel pointed out:

“[Goldstein] was grateful to the country where he and so many others had found asylum first, and a new home – but it was still a home in exile. When he appreciated things American, or criticized them, it was always as an outsider, a spectator. […] It was the American experience that he lacked. In part, I think, it was also a difference of generations. Most of the ‘Americans’ of his acquaintance were a generation or two younger, and the difference in experience was historical as much as geographical. For example, he would often comment on the lack of tradition on this side of the Atlantic. I remember once replying that all the tradition in the world would not help anyone to even the tiniest hamburger, be it here or in Europe. His immediate reply was ‘Ach was,’ followed by ‘The younger generation thinks only of its stomach,’ and, finally by ‘You are probably right, and that is just what is so awful.’ I never could argue him out of that final adjective” (Simmel, 1968, p. 9f).

Although Goldstein tried whatever he could to re-establish a fruitful intellectual exchange with his brother-in-law Cassirer in New York, his Postdoc, the experimental psychologist Martin Scherer (1900–1961), the Cambridge education scholar Robert Ulrich (1890–1977), and the phenomenologist Aron Grutsch (1901–1973), this effort only achieved some ground in physical therapy and rehabilitative psychology in the US (Ulrich, 1968, p. 15).

## 5 Geschwind’s and his pupils’ development of a dynamic localizational perspective

One of his America-based peers who wondered about Goldstein’s stances on holistic neurology and language neuropsychology was Norman Geschwind. He had pursued his initial neurology training at Queen Square (the National Hospital for Nervous Diseases in London, England) through a Mosely Jr. Traveling Fellowship and completed his training under Derek Denny-Brown (1901–1981) at Boston City Hospital as a chief resident. Thus, he can be considered to have been under the sway of the anti-localizationist approaches to aphasia that were forcefully promulgated through the neurophysiological research by British neurologist Henry Head (1861–1940) and others. Interestingly, his first academic appointment was to study muscle neurophysiology at the Massachusetts Institute of Technology before he became interested in aphasia and behavioral neurology. That occurred when he assumed an adjunct neurology faculty position at the Boston University School of Medicine with a primary appointment at the Boston Veterans Administration Hospital. There, he began evaluating patients with focal brain lesions and realized that the classical neurology underlying aphasic deficits was relatively robust and, based on that, he diverted from some of the holistic concepts of brain-behavior relationships (Kushner, 2015, pp. 176–178).

Geschwind helped found North American aphasiology by emphasizing that “every behavior has an anatomy” in the brain (Geschwind, 1964, pp. 220). Geschwind’s method was simple but meticulous, being based on thorough observations of specific behavior, including deficits, described in maximum detail. After identifying the brain lesions, either in imaging approaches of the brain or at the autopsy table, he argued, scientists should correlate the observed behaviors and extent of brain damage; and only then draw their conclusions. His publication on *Language, Behavior, and Epilepsy* speculated on the neuronal correlates of specific behaviors regarding localized brain areas:

“It should be noted that few of them the new students of higher cognitive functions, including [Joseph Jules] Dejerine [1849 -1917], [Hugo Karl] Liepmann [1863-1925], and Goldstein were excessively localizationist as is often assumed – in fact, some of them were less localizationist than Goldstein in relation to their interpretation of many issues! Goldstein stressed more than his predecessors the reaction of normal parts of the nervous system to illness in one portion and highlighted the importance of looking at more than the obvious impairment of the patient” (Geschwind, 1997, p. 60).

His appraisal was directed at the tendency in contemporary neuropsychology to venture into extreme forms of differentiation of the brain into areas, subareas, and centers. Clinical observations in a large variety of individual neuropsychological patients instead seemed to show that phenomenologically oriented differentiation was much greater than was assumed even by the most ardent believers in brain localizationism. Geschwind expressed that the received view in the history of aphasiology held that classical localizationism, based on the structural pathomorphological work of physician Paul Broca (1824–1880) in Paris, France, and Wernicke in Breslau, Germany, gradually began to change during the 1920s. This was a consequence of the efforts by clinically oriented reformers, notably neurologist Pierre Marie (1853–1940) in France, neuroanatomist Konstantin von Monakow (1853–1930) in Switzerland, and neurologist neurologist Head in England. They moved away from “this inadequate classical scheme” and proposed more refined vistas in the field of language disorders (Geschwind, 1964, p. 223). Their revised concepts rapidly achieved a state of domination in the Anglo-Saxon neuropsychology field. Indeed, Geschwind described his own introduction to the traditional localizationist views and their adoption as a cause of personal vexation: “By 1961 I found myself perplexed by the general rejection of anatomical approaches in contemporary writings contrasted with their support by so many of the great classical neurologists. I therefore decided to study the ideas of the classical ‘localizationist’ school by reading their own writings rather than by reading the interpretations of later hostile authors. It was my intention to decide for myself whether the repudiation of the classical views was indeed justified” (Geschwind, 1974, p. 1). On the basis of Geschwind’s clinical and behavioral insights gained from his patients, he expressed that the reform group of the 1920s, including Goldstein, developed schemes of cortical lesions that were not much different from those of their predecessors. Geschwind further deplored that the localizationists in language psychology and aphasiology had not received the attention they deserved (Geschwind, 1964, p. 216)—a view recently repeated by Dutch psycholinguist Pim Levelt in his widely lauded book, *A History of Psycholinguistics. The Pre-Chomskyan Era*:

“It was Henry Head who, in 1926, coined the term ‘diagram maker’ in his fierce attack on the German localizationist school … Where Broca still exclusively used extensive verbal descriptions to present his autopsy results, Wernicke began drawing schematic diagrams of the brain. But the really innovative idea was to draw the functional architecture underlying the psychological skills, to decompose them into their component processes so as to visualize them. Such diagrams are theoretical conjectures that are—or should be—subject to empirical verification. Although this approach has had its ups and downs in the history of psychology, it now belongs to the basic toolkit of any cognitive scientist” (Levelt, 2013, p. 79).

The kind of interdisciplinarity cooperation, as conceived by Head’s “diagram makers,” regarded the communication processes between several related fields as underexplored. They would have to be independently examined based on physiological functions, behavioral analysis, and neuropsychological applications (Carroll, 1951, p. 7).

An example for this call to action can be found in Wernicke’s foundational 19th-century publications in which he developed a modern model of language based on spatially distinct cerebral centers that formed the morphological substrate for the respective nerve actions to process speech and other higher cognitive functions in the brain (Bogousslavsky et al., 2019, pp. 5–6). Specifically in that text, *The Symptom Complex of Aphasia: A Psychological Study on Anatomical Basis* (Wernicke, 1874), Wernicke described his views of language centers being based on a functional neuroanatomy that is quite similar to a ‘parallel distributed neural network’ *avant la lettre* (Eggert, 1977, pp. 22–25) of speech production, spoken word repetition, and word comprehension. In the first part of his groundbreaking work, Wernicke detailed a general theoretical account of neuromorphologically based psychological reflexes which followed along the lines of the work of German-Austrian neuropathologist Theodor Hermann Meynert (1833–1892). In the second part, he offered his own attempts at various abstract diagrams that were seen to represent word production, comprehension, and repetition in various aphasic conditions. In the third part, he reviewed the aphasic symptomatology of ten selected patient cases which he put forward as evidence for this new model (*Zur Unterstuetzung anatomischer Verhaeltnisse*). Four cases had been carefully studied through *postmortem* dissections of their diseased brains (e.g., Wernicke, 1874, pp. 71–73). At the center of Wernicke’s (1874) monograph lay the idea of “psychological reflex archs” based on earlier physiological and neurological theories and contributions that emerged in the 19th century (Canguilhem, 1955, p. 172). These were described in his anthology, *Collected Works and Critical Essays on the Pathology of the Nervous System* (Wernicke, 1893, pp. 1–70). Of particular importance was his connection of psychological reflex archs to subcortical connections—something which would be referred to as neural networks in the present day (Roelofs, 2024, pp. 3–5). The reflex archs coupled the auditory representation of a word in the superior temporal gyrus of the left hemisphere with the movement representation in the inferior frontal gyrus of the same hemisphere through interconnection of the insula. Furthermore, the concept-derived production of words was seen by him as an interplay of auditory, visual, and even taste and olfactory nerve actions (*Nerventaetigkeiten*). Word comprehension itself was conceived as being instantiated through the association of auditory and sensory representations that formed the corresponding concepts (in *Sprechvorstellungen* and *Bewegungsvorstellungen*; Wernicke, 1874, p. 6).

Wernicke derived his insights from earlier criticisms of French experimental physiologist Marie Jean Pierre Flourens’ (1794–1867) theory of the equipotentiality of brain centers in their contribution to human consciousness. Moreover, he deliberately aimed at applying Meynerts’ cerebral fiber theory for practical clinical purposes (*praktisch zu verwerthen*) in order to better understand the normal process of speaking as well as the aphasic disorders in neurological patients (Walusinski et al., 2019, pp. 195–196). His theoretical account of localized centers of language production and control, like Goldstein’s own model, was nonetheless complex and based on several anatomical insights and assumptions on the nerve fiber distribution in the human cortex, as well as between subcortical centers that connected sensory and motor areas (as *Faserungslehre*) (Wernicke, 1893, p. 1). It was able to predict certain forms of aphasic syndromes as well, something which in Wernicke’s time had not yet been described by other neurologists and psychologists. Indeed, many of his contributions, initially overlooked, were gradually rediscovered and expanded upon by researchers and clinicians throughout the 20th century (Ross, 2010, p. 223). It is noteworthy in our present context that Wernicke’s interpretation of the symptom complex of aphasia was accepted in research contributions by Geschwind, his colleagues, and pupils who put forward a similar yet transcending model. This included Wernicke’s emphasis on the crucial role played by the angular gyrus in representing concepts in conjunction with auditory word forms and the idea that naming seemed to proceed solely via auditory word images (Geschwind, 1970, pp. 940–944).

The historic divide between localizationists like Wernicke and holists like Goldstein remained extensive before more recent interdisciplinary endeavors helped to bridge it; specific attention must be drawn here to the “Wernicke-Lichtheim-Geschwind model” (Tremblay and Dick, 2016, pp. 63–65). Among this revisionary and synthesizing trend in neuropsychology were many of Geschwind’s own collaborators and disciples who did their clinical neurology training under him. These included José M. Segarra who worked at the Boston Veterans Administration Hospital (Meadows, 1959, p. 656). Elliott D. Ross received his residency training under Geschwind at Boston City Hospital, as well as a three-month rotation at the Boston Veterans Administration Aphasia Unit at the time when behavioral neurologist D. Frank Benson (1928–1996) served as its director (Cumming, 2010, p. 3). After several neurology positions in the Midwest, he eventually became a faculty member at the University of Oklahoma Health Sciences Center. Among the wider group were the clinical neurologists Antonio Damasio from Portugal, Andrew Kertesz who went to the University of Western Ontario in Canada as well as Michael P. Alexander and Margaret A. Naeser who both continued their research careers at Boston University (Damasio, 1985, p. 391). M.-Marsel Mesulam’s initial academic appointment was with Harvard’s Department of Neurology at the Boston Beth Israel Hospital, under Professor Geschwind; and he later moved to Northwestern University’s Feinberg School of Medicine after Geschwind’s untimely death. As a research group, these figures were able to resolve some of the conundrums of the tension between cerebral localization and broader behavioral features within the context of what Geschwind called the disconnexion syndromes. This work developed over a period of about two decades, offering distinct, challenging, and testable neuropsychological hypotheses (Mesulam, 2015, p. 2795). For example, they established that aphasias could be the result of strictly subcortical lesions while simultaneously documenting the neurological processes that undergirded the recovery of language functions (Mesulam et al., 2009, pp. 2560–2562).

Berlin émigré Fred Quadfasel (1902–1981) had initially familiarized Geschwind with the German school of functional-anatomic relationships as the latter noted in his seminal articles on the disconnexion syndrome published in the international journal *Brain* (Geschwind, 1965a, p. 237; Geschwind, 1965b, p. 585). Ensuing publications by Alexander and LoVerme (1980), Damasio et al. (1982), and Naeser et al. (1987) used computer tomographic scans to show definitively that isolated subcortical lesions, such as in the basal ganglia in the diencephalon, the telencephalic putamen, and parts of the insular cortex and capsula could produce aphasias. Going beyond clinical and pathological descriptions and examinations, recovery of function was studied by Kertesz and colleagues (Kertesz, 1979, pp. 224–230; Kertesz et al., 1993). Likewise, Naeser et al. chaperoned this physiological and behavioral research trajectory when they related the size of morphological lesions involving Wernicke’s cortical area to functional recovery (~ the smaller the lesion, the better the recovery process)—similar to Jay P. Mohr’s (Mohr, 1976) work involving lesions in Broca, 1865 area which affected language fluency (~ the smaller the lesion there, the better the recovery process too) (Naeser et al., 1987). Their contributions laid important groundwork not merely for an increasing understanding of the morphology and pathophysiology of disconnexion syndromes but also for reinterpretations of functional aspects of neuroanatomy and for their connection-related and behavioral foundations as well. Based on this foundational work, other groups of researchers—not directly related to Geschwind and his group at the Veterans Administration in Boston—made further advances into distributed networks that follow Wernicke’s original 19th-century understandings while adding new insights into cortical modulations of sensory architectures and offering predictive qualities (Mesulam, 2015, p. 2797).

The neurologist Mohr at Harvard University’s Massachusetts General Hospital and his colleagues likewise comported themselves clinically like holists and remained antagonistic towards Geschwind’s and Benson’s localizationist approaches to language processing (Mohr, 1976, pp. 201-203). Their findings regarding the role of Broca’s cortical area in speech initiated important dynamic and multi-dimensional brain localization views found in various neurology, psychology, and neuroimaging applications today. Eventually, Ross argued that localization of higher brain functioning needed to be seen as a “very dynamic, four-dimensional phenomenon that is driven by large-scale neural networks learning over time how to efficiently maximize the storage, processing, and modulation of distributed information for cognitive and behavioral operations” (Ross, 2010, p. 233). The underlying large-scale networks, thus, should be conceived in terms of their (1) functional anatomical implications, (2) stimulatory properties, (3) tractography features, and (4) cytoarchitectonic connectivity. Ross’ assumptions were based in large part on a close reading of Wernicke’s theoretical concepts and his traditional functional anatomical correlations in patients with focal brain injuries.

## 6 Discussion

In this essay, we have recalled Geschwind’s exploratory question about a dominant, mostly German-speaking group of scholars that studied aphasia and neuropsychological disorders primarily from a structural point of view. With their work during the latter decades of the 19th century, they related aphasia to a narrowly understood morphological substrate. Yet as Geschwind highlighted, the development of more sophisticated research of language disorders emanated with a group of reform clinicians, including Goldstein, during WWI. In their contributions they reassessed the signs and symptoms of aphasia in close alignment with psychological observations, leading to a meticulous interpretation of the size, scope, and quality of their patients’ cerebral lesions (Geschwind, 1964, p. 224). Geschwind particularly assessed Goldstein’s research on aphasic categorization and the neuropsychology of language, “as a brilliant extension of the works of his illustrious predecessors,” when drawing attention to the latter’s use of clinical aphasic symptoms as diagnostic insights into brain injuries. According to Geschwind, Goldstein’s “holistic theoretical views were [nonetheless] so extensively qualified as to make them compatible with almost any approach” (*Ibid.*, p. 225). His emphasis on there being some discussion on the reasons for the rejection of traditional views in neuropsychology brings us somewhat full circle here (Eling, 2011, p. 504). Even their strongest critics acknowledged multiple insights from the previous localizationist schools.

**Figure 5:**
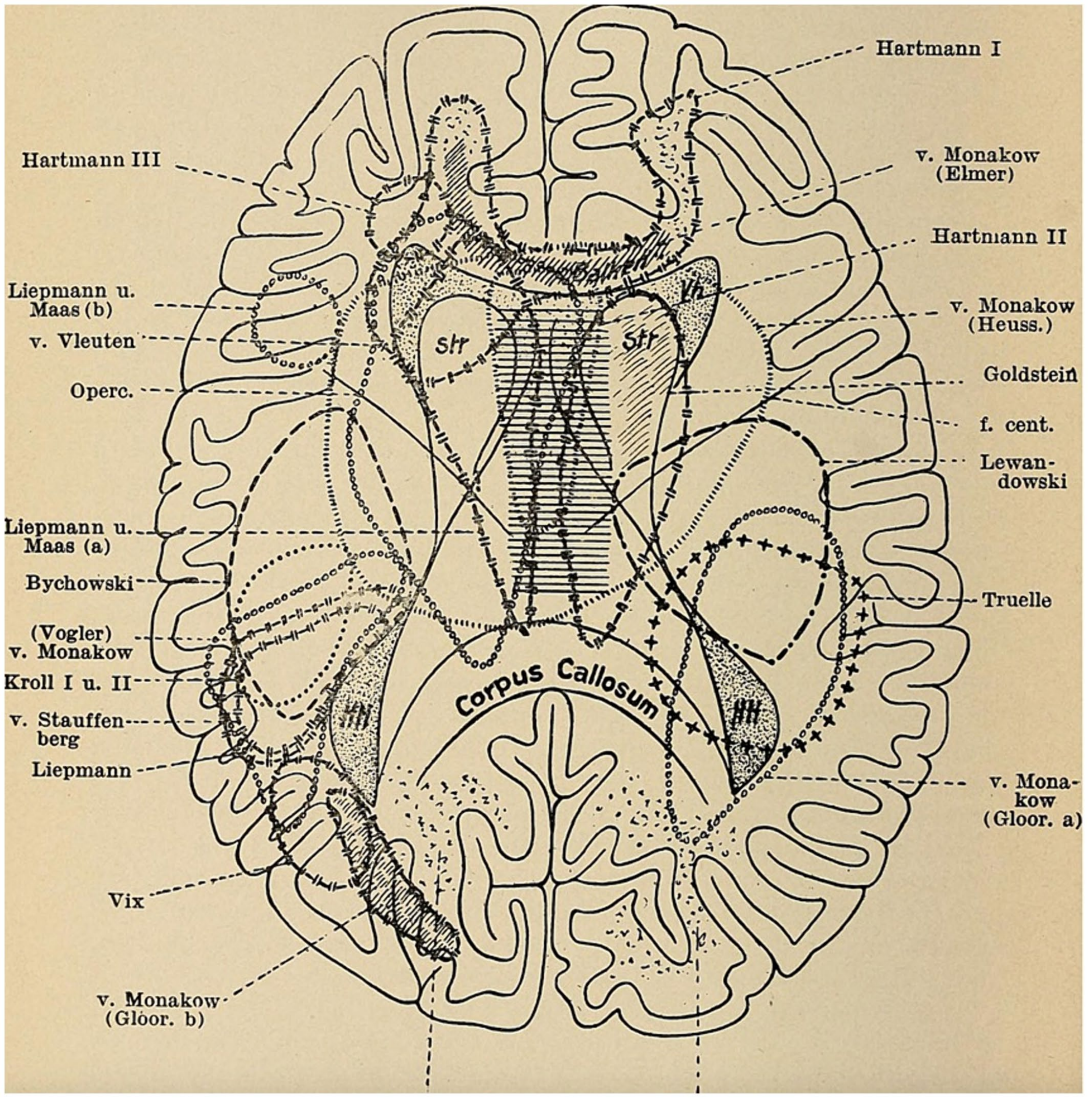
Roeder (1950), *Erkrankungen des Nervensystems*, 78. Sketch © Public Domain.

Beginning with Goldstein’s socialization in the multicultural city of Breslau in the last decades of the German Empire, we have followed his research in medicine, philosophy, and psychiatry, noting that this combination predisposed him to taking an interdisciplinary approach to brain and nervous injuries (Stahnisch and Hoffmann, 2010, pp. 283–285). After the end of WWI, owing to the interactions of many individuals from the contemporary brain sciences and related fields including clinical neurology, experimental psychology, and neurophysiology, the Goldstein group engaged in a rehabilitation program that sought to fully reintegrate brain-injured patients into society (Stahnisch, 2020, pp. 122–130). We have seen that the emphasis on restoring psychological and motor functions was contextualized in the cultural context of the post-WWI period and the Weimar Republic. It reflected the multiperspective attitude found in many of Goldstein’s co-workers, including Gelb, Riese, and Bethe, that contributed to an ever-growing field of cortical and tract lesion models of clinical aphasic symptoms (Figure 5). Their multimodal approach to psychophysiological experimentation and neurorehabilitation could have only flourished in a particular milieu that was conducive to interdisciplinary exchanges, an integration of non-medical scholars from psychology and social work, and early rehabilitational views. It also accessed the understandings and practical insights from early ergotherapists, orthopedic instrument makers, and prosthetic engineers. Unfortunately, the forced migration of most of the members of the Goldstein group eventually caused a resiling from this fruitful potential (Stahnisch, 2010, pp. 60–62).

The outcome of the forced migration of German-speaking neurologists and aphasiologists, including Goldstein, Riese, and Stern, was a situation on the other side of the Atlantic that no longer resembled the foundation of their multimodal and integrative research program (Stahnisch, forthcoming). Any North American reception of their contributions, as Geschwind acknowledged, generally occurred in alignment with traditional localizationist approaches, as well as in specialized rehabilitation communities. This was, for example, the case with personality psychologist Gordon Allport, 1897–1967, humanist psychologist Abraham Maslow, 1908–1970, or group therapist Carl Rogers, 1902–1987, all of whom counted among Goldstein’s new peers in the US (Leahey, 2017, pp. 417–452). Nevertheless, the disposed clinical neurologist barely left an imprint on the neurological community of his new host country as Geschwind highlighted. Accordingly, Geschwind was simultaneously insufficient *and* correct in his assessment if only because he framed an incongruent question: Was Goldstein’s holism so generic that it could be compatible with any localizationist approach? At the risk of theoretically oversimplifying it, Geschwind’s question was not addressing any experimental approach, but was about each clinical patient. Professor Teuber of the MIT further captured this ambiguity regarding Goldstein’s neuropsychological stances:

“The incredibly rapid development of our field in the 50’s and 60’s of this 20th century was bound to make Goldstein into an historical figure, seemingly before his time, but history has a curious way of reaching into the present and of replaying half-forgotten themes in the future” (Teuber, 1966, p. 299).

Accordingly, the neurological study of aphasia, language, and perception disorders in the context of their sufferers’ functional reintegration during the interwar and postwar years needs to be an analysis of geographically shifting scholars, research programs, and institutional units. As significant as Goldstein’s legacy is to modern aphasia research in its applied neurorehabilitational context (Eling, 2015, pp. 17–18), it shows how culturally vulnerable the organization of neuroscientific research endeavors can be, especially when wider interdisciplinary traditions collided with a structurally different North American research context (Sigerist, 1934, p. 3).

Despite Geschwind’s ambivalent assessment of Goldstein’s valuable contributions (“the paradoxial position of Kurt Goldstein”). I intended here to emphasize Goldstein’s neurological concepts, which he derived from a decidedly clinical perspective that focused on the problem of each individual, as a theoretical and clinical bridge between holism and localizationism. This holds true, in particular, when Goldstein’s and Gelb’s assessments of the “disorders of Gestalt” are considered, being representative of the tension around Geschwind’s stances. Such assessments they interpreted both from a psychological and morphological perspective in order not to lose sight of different anatomical instantiations and plastic capabilites of the brain, using different functional perspectives in the concrete situation of neurorehabilitation (Goldstein and Gelb, 1918, p. 124). The nervous system was understood by Goldstein as a functional whole rather than divisible into distinct physiological submodules. Cytoarchitectonical studies suggested functional differences but did not determine them as “neuronal configurations,” instead stressing the importance of “brain plasticity” (Bethe, 1925, pp. 1175–1200). Yet, what Geschwind regarded as Goldstein’s ambivalent position between modern holistic interpretations and traditional localizationist approaches could also be explained through the latter’s clinical recourse to diagnostic analyses of neurological symptoms. Much of this ambivalence can be attributed to Goldstein’s work on the neurorehabilitation of aphasic patients after traumatic war injuries, suggesting that brain functions (even in adults) were plastic (thus, holistic). This, in turn, implied that the strict localization of brain functions, such as language, was incorrect. For practical clinical purposes, Goldstein had intriguingly designed the schemata for clinical diagnoses of head and brain injuries (Goldstein, 1916) into which were incorporated the localization(s) of head injuries in the war-injured veterans he attended (Figure 4). The insights gained thereby were augmented through his ongoing analyses of the clinical behaviors and rehabilitational performance of his patients. Goldstein was even revising his original diagnostic assessments many years after having worked with his patients in Frankfurt and Berlin as can be gleaned from his only return to Germany with the singular purpose of re-examining his former patient, Schneider, at an Oberursel rehabilitation sanatorium (Marotta and Behrmann, 2004, pp. 633–638).

What conclusions can be drawn from the “paradoxical position in the history of aphasia” of Goldstein, neither a holist nor a strict localizationist as Geschwind asserted? Certainly, Goldstein was and still is a monumental figure in neuropsychology, holistic neurology, and the field of neurorehabilitation (Teuber, 1966, p. 299). Interestingly enough, Geschwind remained ambivalent over his elder peer in neuropsychology as was attested by his former pupils from the Veterans Administration in Boston (Kushner, 2015, pp. 173–192). Despite criticisms, Goldstein provided the notion of the “catastrophic reactions” in his neurological follow-up assessments in both the neurological ward and his theoretical account of the observed phenomena. This grants him a mediating place between localizationism and holism that can still be seen as productive in neuropsychological practice (Joswig and Hildebrandt, 2017, pp. 1179–1180):

“But his contribution was not totally to destroy his predecessors, to abolish the diagrams, or to bring order into the chaos. Few scientists create revolutions and the revolution in aphasia occurred in the 1860’s with Broca and in the 1870’s with Wernicke. Most people who advance their field must disagree with their predecessors to some extent and in some measure destroy the past; they must also disagree with their contemporaries and so increase chaos. Usually, a field is least usefully active when it is apparently least chaotic” (Geschwind, 1964, p. 222).

This assessment appears much in line with Teuber’s intriguing view of the achronality of much research in science and medicine (Teuber, 1966, p. 299); historical contributions, such as we see in Goldstein’s early 20th-century case, became of growing interest in the development of the field of neuropsychology and aphasiology in North America during the latter part of the 20th century. Hence, the framework of relearning what has been ignored, or lost in large parts due to political conflict and displacement in neuropsychology throughout the 20th century, is exactly what is at the crux of this article. Indeed, history has a very “curious way of reaching into the present and of replaying half-forgotten themes in the future”—a future in which culturally widespread narratives regarding history determine the interpretation of facts in a particular way (Bruner, 1996, p. 17). Such a process makes Goldstein into a pioneering, though ambivalent, figure in the neuropsychology of aphasia and the wider field of neurorehabilitation.

## Author contributions

FWS: Writing – original draft, Writing – review & editing.
